# Use of acute care services by adults with a migrant background: a secondary analysis of a EurOOHnet survey

**DOI:** 10.1186/s12875-021-01460-6

**Published:** 2021-06-21

**Authors:** Ellen Keizer, Oliver Senn, Morten Bondo Christensen, Linda Huibers

**Affiliations:** 1 Institute of Primary Care, University of Zurich and University Hospital of Zurich, Pestalozzistrasse 24, Zurich, 8091 Switzerland; 2 Research Unit for General Practice, Bartholins Alle 2, Aarhus, 8000 Denmark

**Keywords:** After-hours care, Emergency medical services, Primary health care, Help-seeking behavior, Migrants

## Abstract

**Background:**

High demands create pressure on acute care services, such as emergency medical services (EMS), emergency departments (ED) and out-of-hours primary care (OOH-PC) services. A variety of patient- and organisational factors have been discussed as reasons why especially non-western migrants more frequently contact an ED or OOH-PC service than native born. We aim to investigate whether persons with a non-western and western migrant background more often contact an acute care service than native born and how this relates to the number of contacts with their general practitioners (GPs). In addition, we aim to explore how possible differences in acute care use by migrants can be explained.

**Methods:**

We performed secondary analysis of data collected for the EurOOHnet survey on OOH help-seeking behaviour in Denmark, the Netherlands and Switzerland. Differences in self-reported acute care use (sum of number of contacts with OOH-PC, the ED and 1–1-2/1–4-4) between non-western and western migrants and native born were tested with a quasi Poisson regression analysis. Mediation analyses were performed to examine the impact of factors related to help-seeking on the relation between self-reported acute care use and migrant background.

**Results:**

Non-western migrants had more acute care contacts than native born (adjusted IRR 1.74, 95% CI 1.33–2.25), whereas no differences were found between western migrants and native born. Migrants who regularly contacted OOH-PC or the ED also regularly contacted their GP. Mediation analyses showed that the factors employment, anxiety, attitude towards use of OOH-PC and problems in accessing the own GP could partly explain the higher acute care use of non-western migrants.

**Conclusion:**

The higher use of acute care services by non-western migrants compared with native born could partly be explained by feeling fewer barriers to contact these services, feeling more anxiety, more unemployment and problems making an appointment with the GP. Increasing awareness and improving GP access could help migrants in navigating the healthcare system.

## Background

High demands create pressure on acute care services, such as emergency medical services (EMS), emergency departments (EDs) and out-of-hours primary care (OOH-PC) services [1]. Migrants, especially non-western migrants, are more likely to contact an ED, hospital or OOH-PC service than non-migrants [2–5]. Besides, many studies showed that migrants are more often assessed as presenting non-urgent conditions at the ED than non-migrants [3]. In a previous study we found that persons with a non-western migrant background are more intended to OOH help-seeking than native born [6]. However, variation between studies and countries exists concerning migrants’ use of primary care services compared to native born, both inside and outside office hours [2, 7, 8].

Previous studies have suggested a range of explanations for the higher use of acute care services by migrants, some related to barriers in the healthcare system. Migrants perceive more problems with accessibility to primary healthcare services, mostly with their own general practitioner (GP) [9]. In general, they also seem to have limited knowledge about the healthcare system and the purpose of acute care services [9–13]. For example, in many European countries, the GP acts as gatekeeper, being the first person to contact when experiencing medical problems. Migrants are not always aware of the necessity to contact the GP first, because gatekeeping systems in their country of origin may be non-existing and emergency and specialist care can be accessed directly [2, 14]. Therefore, they could have different ideas of the use of OOH care [15]. Migrants could also have a different perception of urgency or assessment of need for care [16]. Higher morbidity and mortality rates from infectious diseases in non-western countries could lead to a difference in urgency perception of certain health problems [17]. Besides, cultural perspectives of diseases and illness determine healthcare behaviour [18, 19]. Newly arrived migrants generally have a better health status than native born and migrants who arrived a longer time ago, which is often called the health migrant effect [20, 21]. However, over time, their health status decreases, due to several factors such as a lower socio-economic status and perceived discrimination of being a migrant [7, 20, 22, 23].

Healthcare systems aim to provide equitable and patient-centred care for all citizens, making it important to support and improve care for migrants. To increase the equity of care, all citizens need to know how to navigate the healthcare system and to choose the most suitable healthcare provider. Instead of visiting the ED or OOH-PC services, contacting a GP during regular office hours can be a more suitable choice, at least from a medical and cost perspective. A greater understanding of the use of different healthcare services by various patients groups may help to find adequate strategies for interventions. In our study, we aim to answer the following questions:

- Do migrants more often contact an acute care service than native borns?
- How does this relate to the number of contacts with their GP?
- How can possible differences in acute care use by migrants be explained?

## Methods

### Design and population

We performed secondary analysis of data collected for the European Research Network for Out-of-Hours Primary Healthcare (EurOOHnet) survey on OOH help-seeking behaviour in Denmark, the Netherlands and Switzerland [6, 24–26]. The larger study aimed to examine differences in help-seeking between the three countries and to identify factors that are associated with OOH help-seeking. Therefore, we conducted a survey in the three countries in December 2015. The survey was available in the most spoken language of each country (i.e. Danish, Dutch and German).

In line with the overall aim of the primary study, individuals of three age groups were invited to participate in the survey (i.e. parents/caregivers of children 0–4 years, adults 30–39 years and adults 50–59 years). For this present study, we included the two adult age groups. Based on the power calculation for the primary study to detect differences between countries, we aimed to include 600 respondents per country [24]. In Denmark, the Danish Civil Registration System was used to randomly select 1,200 individuals, who received a postal invitation to fill in the questionnaire. The Dutch and Swiss samples were selected using representative consumer panels (i.e. TNS Nipo in the Netherlands; Respondi and Bilendi in Switzerland) [27–29]. in total, 950 Dutch members and 6,093 German-speaking Swiss members of the consumer panels were invited to participate. The different group sizes reflected the different response strategies of the consumer panels.

### Setting per country

In Denmark and the Netherlands, each patient has to be registered at a general practice of his own choice, with GPs acting as gatekeepers for secondary care. Outside office hours, patients can contact a GP cooperative by phone. A visit to the ED is possible, but it is highly recommended to first contact a GP. An ED visit is free of charge in Denmark, whereas Dutch residents must pay an annual tax-deductible fee of at least EUR 375 (2015 figures). Danish and Dutch residents can call 1–1-2 for an ambulance.

In Switzerland, patients can visit all kind of services without referral. However, in return for lower premium costs, they can choose an alternative healthcare plan, which obligates patients to first contact a gatekeeper (i.e. GP or telephone hotline) [25]. The organisation of OOH care varies a lot between regions. Swiss residents can call 1–4-4 when in need of an ambulance. For both GP and emergency care, swiss residents have to pay an annual tax-deductible fee of at least CHF 300 (approx. EUR 275) and 10% co-payment. In all three countries, migrants with residence permit have the same entitlements to GP and emergency care as native born.

### Data collection and questionnaire

A questionnaire was developed consisting of questions on citizen background characteristics, help-seeking (e.g. contacts with healthcare services during the last year) and factors related to help-seeking. The development of the questionnaire is described in detail elsewhere [6, 24]. The English version of the questionnaire is also presented in one of the previously published papers [6]. Factors related to OOH help-seeking were based on Andersen’s Behavioural Model, an acknowledged theoretical framework for individuals’ healthcare use [30].

### Outcome measures

The main outcome measure was the self-reported acute care use in the last year, defined as the sum of the number of contacts with OOH-PC, ED and 1–1-2/1–4-4. Furthermore, we measured the self-reported number of contacts with the own GP in the past year.

The exposure was migrant background, for which we used the definition of Statistics Netherlands: a person of whom at least one of the parents was born abroad [31]. The respondents were categorised into three groups: native born, western migrants and non-western migrants. Native born were persons whose parents were both born in the study country (i.e. Denmark, the Netherlands or Switzerland). Respondents were considered western migrants if one of the parents was born in Europe (except Turkey), North America, Oceania, Indonesia or Japan. Non-western migrants were persons of whom one of the parents was born in a country other than the western countries mentioned before. If parents were born in different countries, mother’s country of birth was used to determine the migrant background of the respondent.

We included the following factors related to help-seeking: age, sex, education level, medical education, work status, living status, social support, health literacy (scales navigating the system and finding information), self-efficacy, anxiety, attitude towards the use of OOH-PC, degree of severity before contacting an OOH-PC service, travel time to closest OOH-PC service, problems with organising a consultation during the day (because of own work/private appointments, accessibility and availability of the own GP), and self-assessed health. A detailed operationalisation of these factors is described elsewhere [6].

### Analyses

Descriptive statistics were used to describe the characteristics of the population as well as self-reported acute care use (i.e. OOH-PC, ED, 1–1-2/1–4-4) stratified by migration background.

Differences in self-reported acute care use between migrants and native born were tested with a quasi Poisson regression analysis (model A). In a second model, we corrected for age, sex and education level, because we saw some (unexpected) differences between the migrant groups for these variables, which may influence the association between migrant background and self-reported acute care use (model B). The relation between use of the own GP and use of acute care was also tested with quasi Poisson regression analyse (model C). To test for differences in this possible relationship between the migrant groups, we tested the interaction effect of migrant background and contacts with the GP (model D).

To examine whether factors related to help-seeking could explain the possible relation between self-reported acute care use and migrant background, we performed mediation analyses. To conduct mediation analyses, two assumptions have to be met: 1) there is a relation between exposure (migrant background) and outcome (acute care use), 2) there is a relation between exposure (migrant background) and the potential mediator [32]. Therefore, we tested the relation between migrant background and our potential mediators, and conducted mediation analyses for the factors associated with migrant background. For each potentially mediator, we conducted a separate mediation analysis in which we also corrected for age, sex and education level. We used the ‘mediation’ R package [33] to calculate the direct effects, causal mediation effects and the total effects of migrant background on self-reported acute care use. All the estimates were expressed in incidence rate ratio’s (IRRs). We used 1,000 bootstrapped simulations to perform the mediation analyses. All analyses were performed in R version 3.2.0.

## Results

### Population

In total, 3,490 persons filled in the questionnaire. Due to the different recruitment methods in the three countries, we could only calculate national response rates: 44.2% in Denmark (*n* = 1,081), 64.5% in the Netherlands (*n* = 1,225) and 19.4% in Switzerland (*n* = 1,184). In the Netherlands and Switzerland, the data collection ended after reaching the required number of respondents. Of all respondents, 79.1% (*n* = 2,733) was native born, 15.8% (*n* = 547) was western migrant and 5.1% (*n* = 175) non-western migrant. From 35 respondents we could not determine the migrant background. Table 1 shows the characteristics of the study population.

**Table 1:** Description of study population grouped by migrant background (%)

Factors	Categories	Native born (N_max_ = 2,733)^a^	Non-western migrants (N_max_ = 175)^b^	Western migrants (N_max_ = 547)^c^
**Age**	Mean (SD)	46.0 (10.2)	39.2 (9.1)	44.4 (9.9)
	Median (IQR)	51.0 (36–55)	36 (32–50)	40 (35–53.5)
**Sex**	Male	47.5	49.1	40.8
	Female	52.5	50.9	59.2
**Education level**	Low	12.5	13.1	8.1
	Middle	52.6	40.6	52.0
	High	34.9	46.3	39.9
**Medical education**	None	89.6	93.1	91.4
	Some/nurse/doctor	10.4	6.9	8.6
**Employment**	Unemployed	19.1	29.2	22.3
	Employed	80.9	70.8	77.7
**Living status**	Living alone	16.8	11.4	19.0
	Living with another adult	83.2	88.6	81.0
**Social support**	Lacking social support	24.0	36.2	29.7
	Receiving social support	75.9	63.2	70.3
**Health literacy – navigating the system**	Low/middle ability	28.4	28.0	35.1
	High ability	71.6	72.0	64.9
**Health literacy – sufficient information**	Low ability	9.5	8.8	15.5
	High ability	90.5	91.2	84.5
**Self-efficacy**	Low	50.6	56.6	40.1
	High	49.4	43.4	59.9
**Anxiety**	No anxiety	88.8	80.8	85.7
	Anxiety	11.2	19.2	14.3
**Attitude towards use OOH-PC**	Low barrier	36.8	52.4	52.5
	High barrier	63.2	47.6	47.5
**Degree of severity before contacting OOH-PC**	Mean (SD)	8.1 (1.5)	7.8 (1.6)	8.2 (1.7)
	Median (IQR)	8 (7–9)	8 (7–9)	8 (7–10)
**Travel time**	< 15 min	45.7	51.4	56.1
	≥ 15 min	54.3	48.6	43.9
**Problems in contacting GP due to own work or private appointments**	No/few problems	84.0	75.6	84.1
	Some/many problems	16.0	24.4	15.9
**Problems in contacting GP due to accessibility own GP**	No/few problems	78.4	71.4	84.7
	Some/many problems	21.6	28.6	15.3
**Problems in contacting GP due to availability own GP**	No/few problems	84.8	80.7	86.4
	Some/many problems	15.2	19.3	13.6
**Self-assessed health**	Poor	13.7	13.7	13.5
	Good	86.3	86.3	86.5

^a^ Percentage of missing values factors ranged from 0% (age) to 4.9% (travel time)

^b^ Percentage of missing values factors ranged from 0% (age, sex, education, medical education, living status, self-assessed health) to 6.3% (travel time)

^c^ Percentage of missing values factors ranged from 0% (age, sex, employment, living status, health literacy: sufficient information, anxiety, self-assessed health) to 5.7% (attitudes towards use OOH-PC)

### Self-reported acute care use

**Fig. 1:**
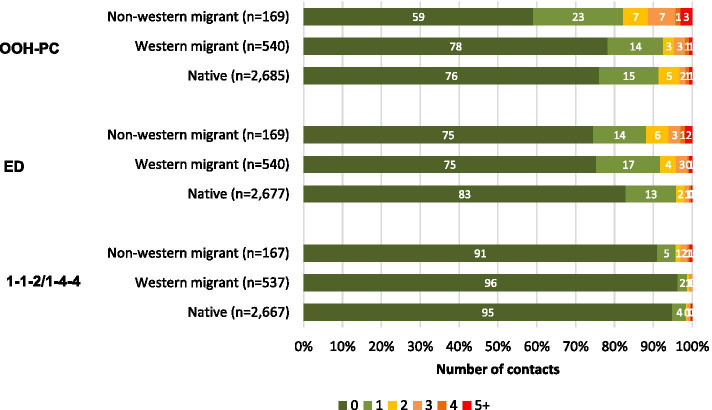
Number of contacts with acute care services grouped by migrant background

Figure 1 shows the number of contacts with the different acute care providers (OOH-PC, ED, 1–1-2/1–1-4), grouped by migrant background. Overall, most of the respondents did not have any contact with an acute care provider. Non-western migrants seem to have more contacts with all acute care providers compared to native born.

We tested the differences in self-reported acute care use between the groups of different migrant background (Table 2). Non-western migrants had more contacts with acute care services than native born (crude IRR 2.02, 95% CI 1.54–2.61; adjusted IRR 1.78, 95% CI 1.35–2.30). We found no significant differences in acute care use between western migrants and native born (adjusted IRR 1.15, 95% CI 0.94–1.40).

**Table 2:** Effect migrant background on acute care use: crude and adjusted (*n* = 2,962)

	**Model A**	**Model B**^**a**^
	**IRR (95% CI)**	**IRR (95% CI)**
Migrant background (ref = native born)		
Western migrant	1.18 (0.96–1.43)	1.15 (0.94–1.40)
Non-western migrant	2.02 (1.54–2.61)*	1.78 (1.35–2.30)*

*ref*  Reference group, *GP*  General practitioner, *IRR*   Incidence rate ratio, *CI*  Confidence interval

^a^ Adjusted for age, sex and education level; *p < 0.05, in bold

### GP care use

**Fig. 2:**
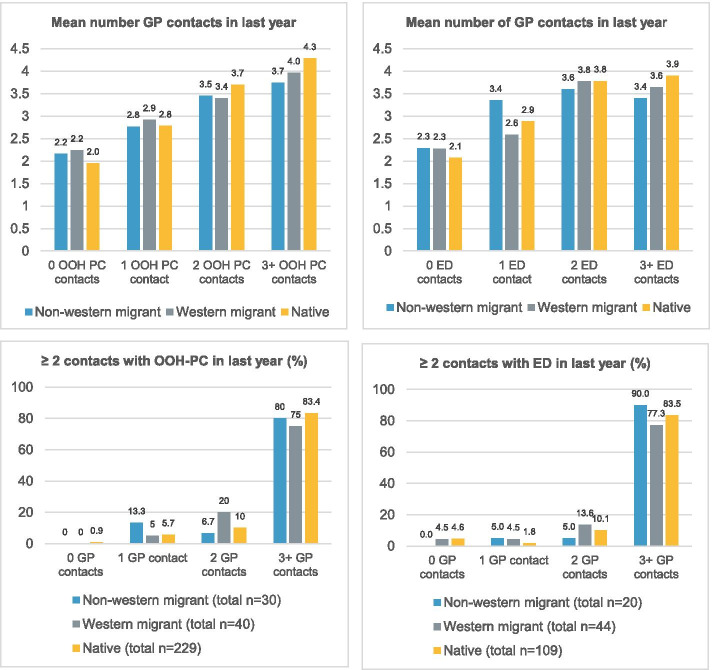
Mean number of contacts with own GP in last year for OOH-PC and ED users

In Fig. 2, we present the mean number of contacts that citizens had with their own GP in the last year stratified by number of OOH-PC contacts and number of ED contacts. For all groups, the mean number of GP contacts seemed to be positively related to the number of OOH-PC contacts and number of ED contacts. Few patients regularly contacted OOH-PC or the ED (≥ 2 contacts) without contacting their own GP in the last year (OOH-PC: non-western migrants 0%, western migrants 0%, native born 0.9%; ED: non-western migrants 0%, western migrants 4.5%, native born 4.6%).

Table 3 show that the number of GP contacts was positively associated with the number of acute care contacts (IRR 1.50, 95% CI 1.44–1.57), and that this effect did not differ between native born and migrants (interaction effect: IRR western migrant: 0.92, 95% CI 0.83–1.02; IRR non-western migrant 0.97, 95% CI 0.83–1.13).

**Table 3:** Influence GP care use on acute care use (*n* = 2,962)

	**Model C**^**a**^	**Model D**^**a**^
	**IRR (95% CI)**	**IRR (95% CI)**
Migrant background (ref = native born)		
Western migrant	1.08 (0.91–1.28)	1.46 (0.97–2.13)
Non-western migrant	**1.54 (1.21–1.93)**	1.73 (0.94–3.04)
Number of GP contacts	**1.50 (1.44–1.57)**	**1.53 (1.46–1.60)**
Interaction		
Western migrant* number of GP contacts		0.92 (0.83–1.02)
Non-western migrant* number of GP contacts		0.97 (0.83–1.13)

*ref* Reference group, *GP* General practitioner, *IRR* Incidence rate ratio, *CI* Confidence interval

^a^ Adjusted for age, sex and education level; *p < 0.05, in bold

### Mediation analyses

**Fig. 3:**
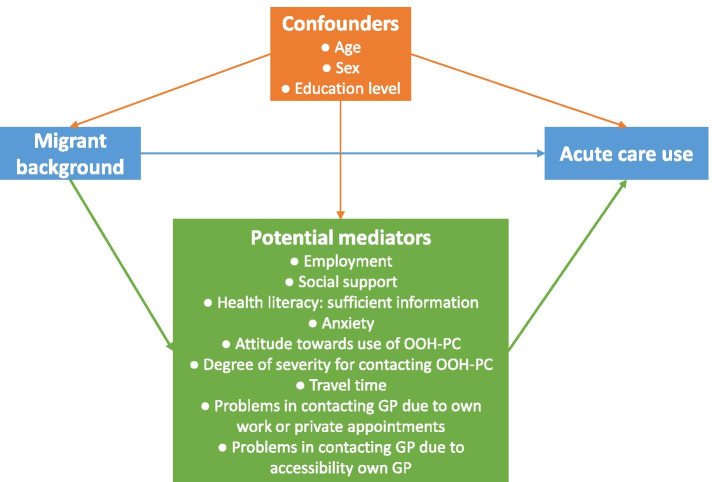
Direct acyclic graph (DAG) mediation analyses

First, we tested whether migrant background was associated with the factors related to help-seeking (potential mediators). The following factors were associated: employment, social support, health literacy: sufficient information, anxiety, attitude towards use of OOH-PC, degree of severity for contacting OOH-PC, travel time and problems in organising a consultation during the day because of own appointments and the accessibility of the own GP (Table 5 in Appendix). Next, we tested whether each individual factor could be a mediation factor in the effect of migrant background on self-reported acute care use (Fig. 3). Since we only found a difference in acute care use between non-western migrants and native born (Table 2), we performed the mediation analyses for these two groups. The incidence risk ratios (IRRs) for the direct, mediation and total effects are shown in Table 4.

**Table 4:** Mediation analyses: effect of migrants background on self-reported acute care use (*n* = 2,962)

Potential mediators	Direct effect	Mediation effect	Total effect	% Mediated
	**IRR (95% CI)**	**IRR (95% CI)**	**IRR (95% CI)**	**% (95% CI)**
Employment	1.69 (1.20–2.51)*	1.04 (1.00–1.09)*	1.75 (1.24–2.64)*	6.9 (0.6–19.0)*
Social support	1.71 (1.21–2.66)*	1.01 (1.00–1.04)	1.74 (1.22–2.66)*	2.2 (-1.0–10)
Health literacy: sufficient information	1.66 (1.20–2.48)*	1.04 (1.00–1.11)	1.72 (1.23–2.61)*	6.8 (-0.3–20.0)
Anxiety	1.65 (1.21–2.46)*	1.05 (1.01–1.11)*	1.73 (1.24–2.61)*	8.4 (1.6–23,0)*
Attitude towards use OOH-PC	1.59 (1.13–2.34)*	1.07 (1.03–1.14)*	1.70 (1.20–2.53)*	13.4 (4.5–35.0)*
Degree of severity before contacting OOH-PC	1.61 (1.16–2.46)*	1.02 (0.98–1.08)	1.65 (1.18–2.56)*	4.6 (-6.3–18.0)
Travel time	1.72 (1.23–2.69)*	0.99 (0.96–1.00)	1.69 (1.21–2.61)*	-2.6 (-11.6–0.0)
Problems in contacting GP due to own work or private appointments	1.67 (1.18–2.56)*	1.03 (1.00–1.07)*	1.72 (1.20–2.69)*	5.8 (0.1–18.0)*
Problems with accessibility own GP	1.68 (1.22–2.56)*	1.02 (1.00–1.06)*	1.72 (1.24–2.59)*	4.2 (0.1–13.0)*

^*^p < 0.05, in bold; all models are adjusted for age, sex and education level

*IRR*  Incidence rate ratio, *CI* Confidence Interval, *GP* General Practitioner, *OOH-PC* Out-of-hours primary care

Attitude toward use of OOH-PC explained 13.4% of the difference in acute care use between non-western migrants and native born (IRR 1.07, 95% CI 1.03–1.14). Furthermore, other factors explained a part of the differences in acute care use between non-western migrants and native born: level of anxiety 8.5% (IRR 1.05, 95% CI 1.01–1.11), employment 6.9% (IRR 1.04, 95% CI 1.00–1.09), problems in organising GP appointments due to own work or private appointments 5.8% (IRR 1.03, 95% CI 1.00–1.07) and problems in accessibility of the GP 4.2% (IRR 1.02, 95% CI 1.00–1.06). The other factors could not explain part of the difference in acute care use between non-western migrants and native born.

## Discussion

### Main findings

Our data shows that persons with a non-western migrant background reported to use more acute care services than native born. We found no difference in self-reported acute care use between persons with a western migrant background and native born. The migrants in our sample who regularly contacted OOH-PC or the ED, also regularly contacted their own GP. Our sample had few migrants who had no contact with their own GP during the past year. Mediation analyses showed that the factors attitude towards use of OOH-PC, anxiety, employment, and problems with organising a GP appointment (due to own work or private appointments and accessibility of the GP) could partly explain the higher acute care use of non-western migrants. Thus, for example, feeling less barriers in contacting OOH-PC care among non-western migrants partly contributes to the higher acute care use by non-western migrants.

### Comparison with literature

Non-western migrants’ higher use of acute care services is also reported in other studies, as is the higher number of contacts for non-urgent problems that could also have been handled by the GP during office hours [3, 34–36]. Migrants’ higher acute care use is often explained by the fact that migrants experience more barriers with access to more appropriate care, such as GP care during office hours [3, 9, 37, 38]. Migrants experience difficulties accessing primary healthcare because of restricted opening hours and long waiting times for making an appointment with the GP [37, 39, 40]. Our study showed that migrants are also able to find their way to their own GP, but these barriers can still be experienced at certain times. Also in our study, the accessibility of the own GP partly explain the higher acute care use of non-western migrants. Some barriers probably mostly concern migrants who have migrated recently and/or experience language barriers [39].

The effect of attitude toward use of healthcare service is hardly investigated. Our mediation analysis showed that non-western migrants felt less barriers in contacting OOH care, which partly explained the higher acute care use. Another study on non-urgent OOH primary contacts found that patients with medically unnecessary problems who contacted an OOH-PC service more often believed that this service is intended for all help requests and not just for urgent requests [41]. The factor anxiety also explains part of non-western migrants’ higher acute care use. Other studies showed that migrants are more likely to experience mental health problems which may explain the higher acute care use. Explanations given for this include labour and economic instability, family separation and racial discrimination [42]. Our analyses showed that unemployment partly explains the higher acute care use by non-western migrants, which is consistent with other studies suggesting that unemployed persons are more likely to use healthcare services [43, 44].

### Strengths and limitations

As far as we know, our study is the first one to examine why persons with a non-western migrant background use more acute care through mediation analyses and the relation with the use of GP care. Therefore, we used a comprehensive overview of relevant factors based on Andersen’s acknowledged behavioural model.

Our study also had some limitations, in particular concerning our sample. Although the proportion of migrants seemed reasonable (20.9% in our sample versus 12.3% in Denmark [45], 21.3% in the Netherlands [46] and 37.2% in Switzerland [47]), the migrants in our sample may not be representative for the migrants in the Danish, Dutch and Swiss populations. Our sample included more highly educated non-western migrants and fewer low educated western migrants than one would expect in a representative sample [48, 49]. Possibly, less educated migrants were less likely to answer the questionnaire and participate in consumer panels, due to language barriers. This bias is probably the result of the research method: a written questionnaire in Danish, Dutch and German. The effects we found could have been stronger if we had included a more representative sample of migrants. Our outcome measure was self-reported acute care use on the last year, which could have introduced recall bias [50]. However, we do not think this has affected our results, since we do not expect differences in accuracy of reporting on acute care use between migrants and native born [51]. We included many factors, some explaining a small part of the higher acute care use of non-western migrants. However, other factors may add an additional explanation of the higher use of non-western migrants, such as language skills, duration of residence in the country of residence, and inflexible working conditions [3]. Besides deficiencies in access to primary healthcare, non-western migrants may have different believes of illness and treatment, and will therefore make different choices in which healthcare provider they contact [6, 15, 39]. Migrants may also experience racism or discrimination and consequently visit another service again for the same problem or make different choices next time, because they or people around them did not feel well helped by certain primary healthcare services [14]. Finally, we are aware that the group of migrants (non-western and western) is very heterogeneous in terms of cultural identity, social situation and help-seeking behaviour [52]. In our paper, we try to draw some general conclusions.

### Recommendations for practice and future research

Our results suggest that the attitude towards use of OOH-PC was important, with persons with a non-western migrant background more often assuming that they have the right to visit acute care services and feeling less barriers to contact them, compared to native born. Thus, education about the purpose of the different acute care services could be useful (e.g. by physicians during consultations, leaflets or meeting for migrants) [15], even as encouraging the use of validated internet tools providing medical information and advice [53]. Frequent attenders could also be identified and invited to discuss considerations for choosing a particular healthcare service. Although non-western migrants also manage to find their way to their own GP, the accessibility of daytime general practice still seems to be a factor in explaining the higher acute care use. Therefore we recommend to study improving the accessibility of daytime general practice for non-western migrants.

A qualitative study would be recommended as a supplement to this study. For example, interviews could be conducted to get insight into the considerations during the decision making process and final choice of a particular acute care service. Using interviewers who speak the language of the migrants could also reach the less well-integrated migrants.

## Conclusion

Non-western migrants make more use of acute care services than native born, while western migrants make as much use as native born. Non-western migrants seem to be able to find their way to their own GP, but may be more likely to go to OOH care services or the ED for the same medical problem than native born. This could partly be explained by feeling fewer barriers to contact these services, feeling more anxiety, more unemployment and problems making an appointment with the GP. Increasing awareness and improving GP access could help non-western migrants in navigating the healthcare system.

## Data Availability

The dataset used in the current study are available from the corresponding author on reasonable request.

## Appendix

Table 5

**Table 5:** Association migrant background and factors related to help-seeking. Non-western migrant versus native born

Factors	Chi-square	*P*-value
Medical education	2.22	0.14
Employment	10.39	< 0.01
Living status	3.46	0.06
Social support	12.94	< 0.01
Health literacy – navigating the system	3.53	0.06
Health literacy – sufficient information	6.75	< 0.01
Self-efficacy	2.38	0.12
Anxiety	10.04	< 0.01
Attitude towards use OOH-PC	16.48	< 0.01
Degree of severity before contacting an OOH-PC service^a^	2.54	0.01
Travel time	6.77	< 0.01
Problems in contacting GP due to own work or private appointments	8.08	< 0.01
Problems in contacting GP due to accessibility own GP	4.45	0.03
Problems in contacting GP due to availability own GP	2.06	0.15
Self-assessed health	< 0.01	0.98

^a^independent t-test was used to test the differences

## Abbreviations

CI: Confidence Interval
DAG: Direct acyclic graph
ED: Emergency department
EMS: Emergency medical services
GP: General practitioner
IQR: Interquartile range
IRR: Incidence rate ratio
OOH: Out-of-hours
OOH-PC: Out-of-hours primary care
SD: Standard deviation
